# Assessing Anxiety Disorders Using Wearable Devices: Challenges and Future Directions

**DOI:** 10.3390/brainsci9030050

**Published:** 2019-03-01

**Authors:** Mohamed Elgendi, Carlo Menon

**Affiliations:** 1 Menrva Research Group, Schools of Mechatronic Systems Engineering and Engineering Science, Simon Fraser University, Surrey, BC V3T 0A3, Canada; 2 School of Electrical and Computer Engineering, University of British Columbia, Vancouver, BC V6T 1Z4, Canada; 3 Faculty of Medicine, University of British Columbia, Vancouver, BC V1Y 1T3, Canada; 4 BC Children’s & Women’s Hospital, Vancouver, BC V6H 3N1, Canada

**Keywords:** digital medicine, anxiety, depression, mental well-being, flourishing, self-help with email support, biosignals, mental health promotion, wearable devices

## Abstract

Wearable devices (WD) are starting to increasingly be used for interventions to promote well-being by reducing anxiety disorders (AD). Electrocardiogram (ECG) signal is one of the most commonly used biosignals for assessing the cardiovascular system as it significantly reflects the activity of the autonomic nervous system during emotional changes. Little is known about the accuracy of using ECG features for detecting ADs. Moreover, during our literature review, a limited number of studies were found that involve ECG collection using WD for promoting mental well-being. Thus, for the sake of validating the reliability of ECG features for detecting anxiety in WD, we screened 1040 articles, and only 22 were considered for our study; specifically 6 on panic, 4 on post-traumatic stress, 4 on generalized anxiety, 3 on social, 3 on mixed, and 2 on obsessive-compulsive anxiety disorder articles. Most experimental studies had controversial results. Upon reviewing each of these papers, it became apparent that the use of ECG features for detecting different types of anxiety is controversial, and the use of ECG-WD is an emerging area of research, with limited evidence suggesting its reliability. Due to the clinical nature of most studies, it is difficult to determine the specific impact of ECG features on detecting ADs, suggesting the need for more robust studies following our proposed recommendations.

## 1. Introduction

Anxiety disorders (AD) are the most common type of mental illness in the world, affecting 264 million worldwide [1]. Every 10 years, there is an increase of AD by 14.9% [2]. Common mental illness such as anxiety and depression are increasing, especially in low- and middle-income countries [3]. These staggering statistics have motivated researchers to develop new and improved technologies to promote well-being and to reduce related morbidity, mortality, and health care costs. Moreover, the gold standard for assessing anxiety and other psychiatric disorders in children or adults is the clinical interview [4]. Attaining an appointment with a mental health consultant is not an easy process that often takes time, and unfortunately by the time an appointment is reached with a psychologist a person’s life can be in danger.

The increase of using wearable devices (WD), such as electrocardiogram (ECG) smartwatches and belts, along with mobile apps, have given new opportunities for WDs to influence our decisions and behaviors. Moreover, the recent advances in analyzing ECG signals for WDs for algorithm development [5,6], detecting R peaks [7], compression [8,9], and visualization [10] create a more reliable technology. WDs can be used at any time and in any environment, enabling objective mental status feedback based on the collected ECG data. In general, WD technologies have been already used in health interventions, providing promising results [11]. However, so far, these interventions have not been adopted for addressing ADs. Thus, integrating wearable ECG sensors (or other sensors) into our clothes, phones, and accessories could help users at risk and could lead to relatively positive health outcomes. Note that in the literature, to our knowledge, there are three measures for assessing autonomic nervous system functioning in emotion: cardiovascular, electrodermal, and respiratory measures [12]. However, the focus of this study is investigating the cardiovascular measures, specifically using ECG signals.

Several reviews investigated the correlation of ECG features with different types of AD; however, these studies were often limited to clinic-based measurement, or a healthcare setting. No review has examined the quality of the ECG signal, the measurement setting, the ECG sensor location, or the portability of the device. Moreover, many papers published ECG features that contradicted with other findings on the same topic [13]. In other words, the ECG features used in some papers to detect the presence or absence of AD in subjects were reported as not used to detect the presence or absence of AD in other papers. For these reasons, limited research has been carried out to assess the validity of ECG and its efficacy and robustness in detecting AD, especially for realtime feedback. Therefore, we sought to highlight the ECG features and their challenges, and provide recommendations towards using ECG effectively with WDs.

## 2. Methods

The search strategy relied on selecting relevant peer-reviewed studies according to PRISMA [14] guidelines, with all relevant combinations of the following words and phrases: ‘ECG features,’ ‘heart rate variability,’ ‘generalized anxiety disorder,’ ‘panic disorder,’ ‘obsessive-compulsive disorder,’ ‘post-traumatic stress disorder,’ ‘social anxiety disorder,’ ‘social phobia,’ ‘specific phobia,’ and ‘wearable.’ In addition to these electronic searches, each report’s citation list was examined for additional studies. The search started in July 2018, with no limitation on the duration of the search. Inclusion criteria were as follows: (1) ECG features were used to detect any type of anxiety (2) ECG features needed to be stated clearly in each publication (3) statistically reliable correlations between ECG features and different types of AD needed to be reported (4) control subjects had to be free from psychiatric disorders. All selected articles were reviewed and adjudicated by the two investigators (ME and CM).

## 3. Results and Discussion

**Figure 1:**
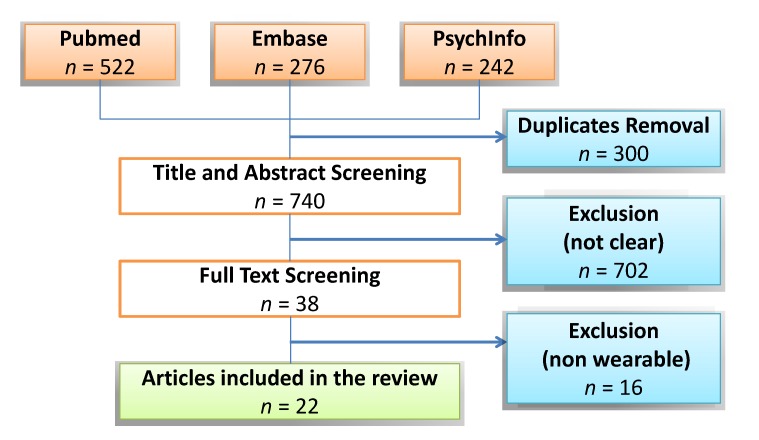
Flow diagram of included studies. Here, 22 studies were identified from 1040 articles in the initial database search (July 2018). Search updates were conducted until Nov 2018.

The database search retrieved 1040 articles, as shown in Figure 1; 300 duplicates were removed. After title and abstract screening, 718 articles were excluded after full-text review, for not meeting inclusion criteria regarding mentioning ECG devices, mentioning ECG features used, unclear outcomes or improper study design. The remaining 22 articles were considered for our study, specifically six on panic, four on post-traumatic stress, four on generalized anxiety, three on social, three on mixed, and two on obsessive-compulsive anxiety disorder articles.

**Figure 2:**
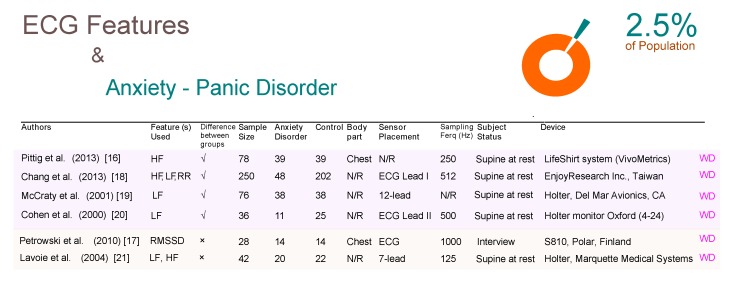
Comparison of ECG-based studies between subjects with and without panic disorders. Abbreviations: ECG, electrocardiogram; RR, R-peak to R-peak in ECG signal; LF, low frequency; HF, high frequency; RMSSD, root mean square standard deviation; WD, wearable device; N/R, not reported.

Our results highlight crucial aspects for developing and designing a WD study for detecting AD using ECG features. For example, Figure 2 shows a comparison between different studies for subjects with and without panic disorder (PD). The table is split vertically into two parts; the above part shows the studies that confirm the efficacy of ECG features for detecting PD while the bottom part shows the studies that reported no significance of ECG features. Most of the studies did not mention the body part used for ECG data collection. All studies were carried out on subjects who are resting on a supine position. One promising WD-based research used a shirt with built-in ECG sensors. In general, PDs have attracted most researchers compared to other ADs as it affects approximately 2.5% of people at some point [15].

As can be seen in Figure 2, there are six studies, all of which used ECG-based WDs. The first four studies demonstrate results that contradict the latter two studies regarding the usability of ECG features. In other words, the ECG features are used to detect the PD in the first four studies, while in contrast in the last two studies no significance was shown for PD detection. Note that the Holter device is considered a WD for the purpose of this paper even though it is not wireless and the data analysis for the device is carried out offline. Sensor placement on the body for each study was inconsistent; four studies used one lead, one study used seven leads, and one study used twelve leads. The two most promising studies, with regards to the WD applicability for detecting PD, is the study published by Pittig et al. [16] and Petrowski et al. [17]. Pittig et al. utilized the Lifeshirt system which is essentially a lightweight, comfortable garment with embedded sensors over a sample size of 78 subjects. Petrowski et al. utilzed the S810 Polar heart rate monitor which is essentially two components (a transmitter around the chest and a receiver around the wrist) over a sample size of 28 subjects. Ironically, these two promising studies presented opposing results; Pittig et al. showed the significance of ECG features for detecting PD, whereas Petrowski et al. did not show significance.

Note, Chang et al. [18], McCraty et al. [19], and Cohen et al. [20] were in agreement that the low frequency is an effective ECG feature to detect PD. On the other hand, Petrowski et al. [17] and Lavoie et al. [21] showed no significance of time- and frequency domain ECG features.

**Figure 3:**
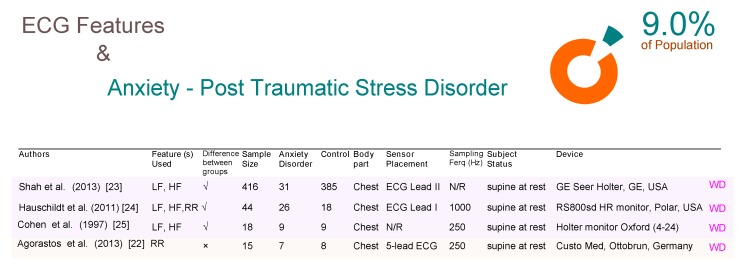
Comparison of ECG-based studies between subjects with and without post traumatic stress disorder. Abbreviations: ECG, electrocardiogram; RR, R-peak to R-peak in ECG signal; LF, low frequency; HF, high frequency; WD, wearable device; N/R, not reported.

Post traumatic stress disorder (PTSD) affects 9% of the world population, and interestingly, seems to be less investigated in comparison to other anxiety disorders such as PD. In Figure 3 there are four ECG-based WD studies, three of which confirm the value of ECG’s role is detecting PTSD, while the fourth paper does not demonstrate this value. Interestingly, the two studies that used a Holter device (which is clinically approved) are in agreement with the polar watch (which is not yet clinically approved) showing that the frequency analysis of ECG signals can help detect PTSD.

Agorastos et al. [22] used the Custo Med device which is essentially a wearable device that utilizes wireless Bluetooth technology for ECG transmission over 15 subjects, which is the smallest sample size among the four studies for PTSD. Additionally, the time domain analysis was used with Agorastos et al. while the other three studies used the frequency domain analysis. It is unclear if the contradiction in results are due using different domain analysis, or in other words, due to different ECG features being used. Note, Shah et al. [23], Hauschildt et al. [24], and Cohen et al. [25] were in agreement that the low frequency is an effective ECG feature to detect PTSD.

**Figure 4:**
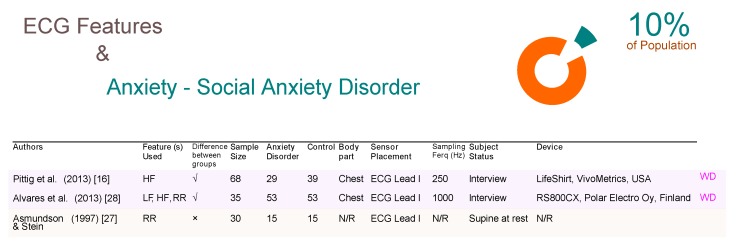
Comparison of ECG-based studies between subjects with and without social anxiety disorders. Abbreviations: ECG, electrocardiogram; RR, R-peak to R-peak in ECG signal; LF, low frequency; HF, high frequency; WD, wearable device; N/R, not reported.

Social anxiety disorder (SAD) affects approximately 10% of the world population and is under investigated in comparison to other anxiety disorders [26]. Two of the three studies in Figure 4 used WD, whereas in the third study they did not report as to whether or not the device used as a WD or not. Given that the study was done in 1997, we can assume that the Asmundson and Stein [27], conducted over a sample size of 30 subjects, was not a WD study. However, we used their article as the results show that ECG signals are not significant for detecting SAD. Pittig et al. and Alvares et al. [28] collected ECG signals from the chest and confirmed the significance of using ECG features for detecting SAD.

**Figure 5:**
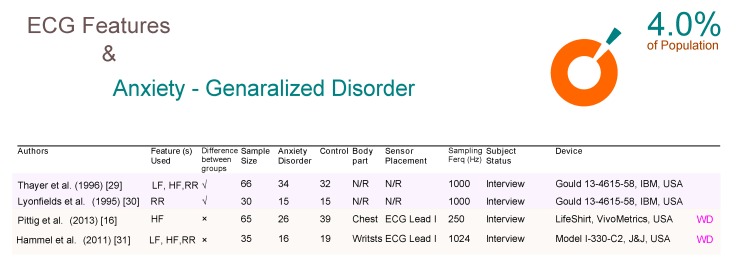
Comparison of ECG-based studies between subjects with and without generalized anxiety disorders. Abbreviations: ECG, electrocardiogram; RR, R-peak to R-peak in ECG signal; LF, low frequency; HF, high frequency; WD, wearable device; N/R, not reported.

Generalized anxiety disorder (GAD) affects 4% of people at some point, [15] and four relevant studies have been included in Figure 5. Unfortunately, none of the four studies confirmed the significance of ECG for detecting GAD. Two of the studies (Thayer et al. [29] and Lyonfields et al. [30]) that used a non-WD technology, and the two WD studies (Pittig et al. [16] and Hammel et al. [31]) confirmed that ECG features are not useful for detecting GAD. These two studies collected the data from two different body measurement sites; one from the chest and the other from the wrist.

**Figure 6:**
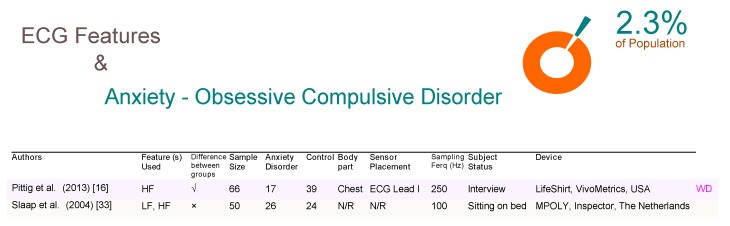
Comparison of ECG-based studies between subjects with and without obsessive compulsive disorder. Abbreviations: ECG, electrocardiogram; LF, low frequency; HF, high frequency; WD, wearable device; N/R, not reported.

As shown in Figure 6, obsessive-compulsive disorders (OCD) affect 2.3% people at some point in their life [32], and only one ECG-based WD study (Pittig et al. [16]) was found that confirms the significance of ECG features in detecting OCD. The other non-WD study (Slaap et al. [33]) showed no significance.

**Figure 7:**
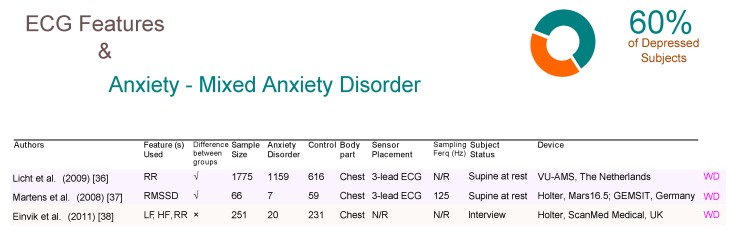
Comparison of ECG-based studies between subjects with and without mixed anxiety disorders. Abbreviations: ECG, electrocardiogram; RR, R-peak to R-peak in ECG signal; LF, low frequency; HF, high frequency; RMSSD, root mean square standard deviation; WD, wearable device; N/R, not reported.

Interestingly, both reported their results using a frequency domain analysis. Mixed anxiety disorder (MAD) affects 60% of depressed people [34,35]. Three ECG-based WD studies were identified, two of which ( Licht et al. [36] and Martens et al. [37]) confirmed that the time domain analysis of ECG features could detect MAD, as shown in Figure 7. An interesting study by Licht et al. used a VU-AMS device, which is essentially an ambulatory monitoring system that consists of seven electrodes over a sample size of 1775 subjects, which is the largest sample size of all studies included in this review. Note that the system was intended to be used a non-invasive device for the measurement of the autonomic nervous system for research. In fact, if we add each of the total sample sizes for each article included in this paper (with the exception of the Licht et al. study), the total would still not amount to the 1775 of the Licht study. Thus, this study alone due to its large subject base, strongly demonstrates the significance of the ECG features for detecting MAD. Interestingly, Licth et al., showed that time domain analysis can be informative and detect MAD. Note, Einvik et al. [38] demonstrated that ECG features are not useful for detecting MAD.

**Figure 8:**
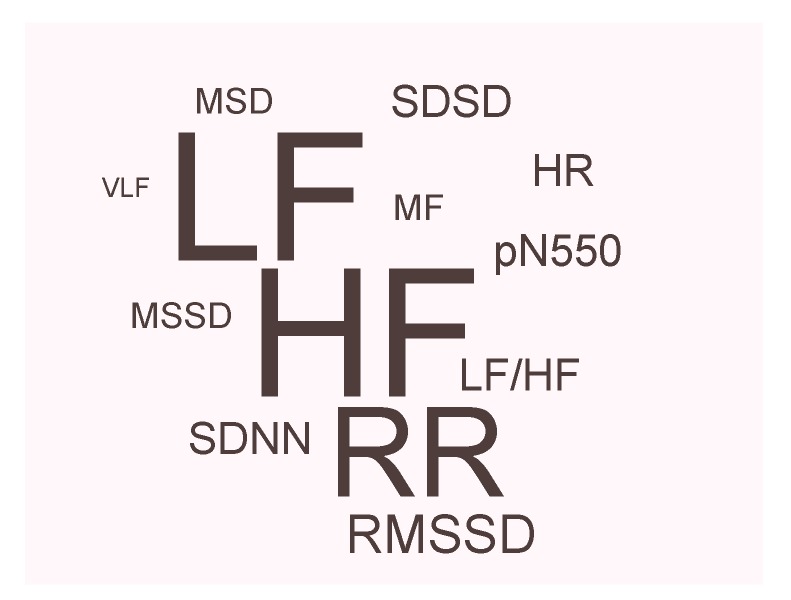
Relative frequency of ECG features used in wearable devices for detecting anxiety disorders.

Figure 8 illustrates of the relative number of WD studies that have used ECG features to detect anxiety disorders. This figure shows that low frequency (LF) and high frequency (HF) are the most often reported ECG features followed by R-peak to R-peak in the ECG signal (RR intervals). Other ECG features were used such as heart rate (HR), mid-frequency spectral (MF), mean difference between successive RR intervals (MSD), mean square of successive RR interval differences (MSSD), root-mean-square of successive RR interval differences (RMSSD), percentage of successive normal sinus RR interval >50 ms (pNN50), standard deviation of the normal-to-normal intervals (SDNN), standard deviation of successive differences (SDSD), and very low frequency spectral (VLF). Note that the LF and HF are the most used ECG features while VLF is the least used feature.

This review has many advantages in the development of ECG-based WDs for detecting anxiety disorders in realtime in the near future. As can be seen in Figure 2, Figure 3, Figure 4, Figure 5, Figure 6 and Figure 7, researchers reported different examination results of ECG features, and here is a breakdown of the inconsistencies that led to this issue:

- Small sample sizes.
- Omission of discussion of confounding factors, such as psychiatric and medical co-morbidity.
- Limited information on subject medication intake status before running the study.
- Collection of ECG signals from different positions such as arm, chest, etc.
- Collection of ECG signals with different ECG devices such as Holter, portable, clinical setting, etc.
- Selection of ECG leads ranged from 1 lead to 12 leads.
- Sampling ECG signals varied from 100–1024 Hz, this may play a significant role in the extraction of frequency-based ECG features.
- Varying environments during the data collection that impacts emotions (e.g., subject laying down in the lab vs. going home).

There is a lack of studies that use ECG-based WDs to detect different anxiety disorders, especially OCD, GAD, and SAD, making difficult to assess the value of using ECG features in detecting these disorders. Only one study reported the significance of using ECG-based WD in detecting OCD. On the other hand, two studies reported no significance of using ECG features for detecting GAD. We also did find longitudinal studies to analyze the ECG features from different anxiety disorders.

It is worth noting, subjects with an anxiety disorder suffer from co-morbid depression in up to 60% of cases [39]. So, it is difficult to differentiate between subjects with and without psychiatric co-morbidity. However, Lichet et al. showed that ECG features can detect the multiple anxiety disorders effect.

We also noticed it is rarely to find a study that discusses treatment based on ECG features assessment, suggesting the need for more studies to confirm the impact of ECG features in treating anxiety disorders. To be more clear, we need more ECG-based WD studies investigating health and well-being. In general, findings from different WD studies show an overall correlation between ECG features and the majority of anxiety disorders. However, it is not clear if the majority of subjects in these articles had cardiovascular diseases or not. Many factors can contribute to the changes in ECG features. Moreover, there is a need for examining the WD intervention for different anxiety disorders and how ECG-based WD can be used to reduce mental illness and improving well-being.

## 4. Future Directions

In the near future, real-time analysis of ECG waveforms using wearable devices may provide a more comprehensive analysis of AD over the course of a day. Thus, wearable devices that can noninvasively detect AD are expected to play an increasingly important role in clinical medicine and outpatient care. The area of detecting (or monitoring) different types of AD using ECG signal is relatively new, and most ECG analysis methods have been relatively simple. As can be seen from the figures, many of the published results that discussed the correlation between ECG features and AD are contradictory, and many of the studies had very small sample sizes. Most researchers used only ECG signals and a few used ECG along with other biosignals. Based on the current literature review of AD detection using ECG signals, we recommend the following:

1. Collect multiple biosignals along with ECG;
2. Ensure that all biosignals are collected at the same time without any delay (checking the time synchronization between all sensors is highly required);
3. Collect biosignals using WDs along with an ECG monitor (preferably FDA approved device and calibrated once every two years) to validate results;
4. Collect biosignals from a large number (>100) of study subjects; pure control, as well as pure AD (without no confounding factors) need to be used for study validation;
5. collect biosignals from a diversity of subjects: young, old, female and males, different skin color and ethnicities;
6. Collect data from subjects under both resting and movement conditions, to validate robustness, in case of developing a wearable device;
7. investigate the correlation of other morphological ECG features (e.g., P wave, Q wave, R wave, S wave, T wave, and their changes in terms of amplitudes, slopes, intervals, energies, entropies, etc.) with AD;
8. Collect a psychological questionnaire such as GAD-7 before/during/after running the study;
9. Organize, if possible, a structured interview with a psychologist to validate results obtained from the previous two points;
10. Produce publicly available physiological databases that contain time-synchronized biosignals. Although some public databases exist, such as Eight-emotion Sentics Data, MIT Affective Computing Group, [40] these data were collected from one subject targeting the eight emotional states: neutral, anger, hate, grief, love, romantic love, joy, and reverence;
11. Encourage closer collaboration between the engineering community and clinical researchers who have access to patients and have experience designing and implementing the validation protocols that are necessary to move forward this type of work.

Perhaps some of the recommendations are intuitive and known to most scientists; however, we emphasized these recommendations as most papers (if not all) did not clearly determine or achieve these recommendations. Clearly outlining recommendations can serve as a clear reminder and also help to avoid misleading and contradictory conclusions.

ECG-based WDs for remote and ambulatory monitoring are becoming an effective solution and increasingly more accepted for monitoring daily activity, exertion and sleep, with implications for home-based care [41]. With the growing adoption of ECG-based WDs, the mental conditions of the large spectrum of users can be continuously monitored. Providing suggestions to the users are triggered when an unusual emotional change is detected. One of the proposed suggestions is an interactive feedback system, such as chatbot, to make suggestions such as ‘did you exercise today?’, ‘when did you last sleep?’, ‘do you feel tired?’, or ‘how about mindfulness, try it for 5 min?’. This feedback feature will be available on an individual’s phone for remote and local assessment, in addition to generating an alarm to alert the emergency contact person if there is a critical situation, and/or the chatbot will call healthcare service providers for help. The ECG-based WDs can enable people with fragile mental states to have increased control over their daily life activities, improve their mental well-being, and assist in avoiding significant levels of anxiety and fear that may lead to hazards or risk. Developing reliable ECG-based WDs for real-time anxiety assessment will increase the level of independence, reduces costs imposed on the healthcare system, and help minimize preventable loss of lives.

## 5. Conclusions

Features extracted from the electrocardiogram (ECG) signal are the primary indicators of well-being status used clinically in humans. The timely detection of anxiety disorder (AD) is of great importance, as early detection and intervention can improve the outcomes of any mental disorder. In this review, we have introduced the current theoretical approaches and wearable applications of noninvasive, AD detection techniques. Currently, most ECG-based AD detection is mainly divided into two research directions based on time- and frequency-domain analysis. Research based on the ECG waveform morphologies is limited. Also, exploring multiple signals may lead to more informative research findings that can enable the implementation of more complex wearable designs. Currently, there are many challenges, such as the sensor type, the sensor location, confounding diseases. Moreover, there is a need for a large amount of data and a certain period of pre-training and calibration.

The development of noninvasive and continuous AD detection (or prediction) is a promising yet challenging field. The trend is toward wearable ECG technology is evident. In future studies, extracting and integrating more ECG features can hopefully enable researchers to solve the above-mentioned problems and to successfully develop technologies for AD estimation using mobile and wearable devices.

## Abbreviations

The following abbreviations are used in this manuscript:

AD	Anxiety disorder
ECG	Electrocardiogram
GAD	Generalized anxiety disorder
HF	High frequency
LF	Low frequency
MAD	Mixed anxiety disorder
OCD	Obsessive-compulsive disorders
PD	Panic disorder
PTSD	Post traumatic stress disorder
RR	R-peak to R-peak in ECG signal
RMSSD	Root mean square standard deviation
SAD	Social anxiety disorder
WD	Wearable device
